# Supplementary material for: Analysis of conflict of interest policies among organizations producing clinical practice guidelines
Source: PLoS One. 2021 Apr 30;16(4):e0249267. doi: 10.1371/journal.pone.0249267 (PMC8087455; doi:10.1371/journal.pone.0249267)
Supplement: S1 Appendix — (DOCX) [file pone.0249267.s001.docx]

**S1 Table. Characteristics, COI policy information, and adherence to IOM standards by organization**

| **Guideline Society** | **Type of Organization** | **Region** | **COI policy (Y/N)** | **Date of Policy** | **No. IOM standards met by policy** |
| --- | --- | --- | --- | --- | --- |
| American Academy of Dermatology | Professional | US | Y | 11/2/2019 | 3 |
| American Academy of Neurology | Professional | US | Y | 6/17/2017 | 4 |
| American Academy of Orthopaedic Surgeons | Professional | US | Y | 4/1/2019 | 3 |
| American College of Cardiology | Professional | US | Y | 8/28/2019 | 4 |
| American College of Chest Physicians | Professional | US | Y | 2/1/2020 | 4 |
| American College of Gastroenterology | Professional | US | N | N/A | N/A |
| American College of Obstetricians and Gynecologists | Professional | US | N | N/A | N/A |
| American College of Radiology | Professional | US | Y | Not dated | 2 |
| American Heart Association | Professional | US | Y | 6/1/2010 | 4 |
| American Society for Gastrointestinal Endoscopy | Professional | US | N | N/A | N/A |
| American Society for Radiation Oncology | Professional | US | Y |  | 5 |
| American Society of Clinical Oncology | Professional | US | Y | 8/8/2013 | 3 |
| American Society of Colon and Rectal Surgeons | Professional | US | N | N/A | N/A |
| American Society of Hematology | Professional | US | N | N/A | N/A |
| American Thoracic Society | Professional | US | Y | 1/7/2015 | 4 |
| American Urological Association | Professional | US | Y | 10/1/2018 | 4 |
| British Association for Sexual Health and HIV | Professional | Europe | Y | Not dated | 7 |
| British Association of Dermatologists | Professional | Europe | Y | 7/1/2017 | 4 |
| British Society of Gastroenterology | Professional | Europe | Y | 12/1/2018 | 3 |
| Canadian Cardiovascular Society | Professional | Canada | Y | 1/1/2019 | 3 |
| Cancer Care Ontario | Government | Canada | Y | 5/15/2018 | 4 |
| Cancer Control Alberta | Government | Canada | Y | 6/1/2019 | 2 |
| Centers for Disease Control and Prevention | Government | US | Y | 10/1/2015 | 2 |
| Cincinnati Children's Hospital Medical Center | Health Care Provider | US | N | N/A | N/A |
| Eastern Association for the Surgery of Trauma | Professional | US | Y | 10/1/2016 | 1 |
| Endocrine Society | Professional | US | Y | Not dated | 6 |
| Enhanced Recovery After Surgery Society | Professional | Europe | N | N/A | N/A |
| European Association of Urology | Professional | Europe | Y | Not dated | 4 |
| European League Against Rheumatism | Professional | Europe | Y | 9/26/2014 | 1 |
| European Society for Paediatric Gastroenterology, Hepatology and Nutrition | Professional | Europe | N | N/A | N/A |
| European Society for Vascular Surgery | Professional | Europe | Y | 9/1/2017 | 2 |
| European Society of Cardiology | Professional | Europe | Y | 12/17/2019 | 1 |
| European Society of Gastrointestinal Endoscopy | Professional | Europe | Y | 1/1/2018 | 0 |
| European Stroke Organization | Professional | Europe | Y | 7/6/2015 | 2 |
| Infectious Diseases Society of America | Professional | US | Y | 2/1/2018 | 4 |
| MAGIC-BMJ | Journal | Europe | Y | Not dated | 6 |
| Ministry of Public Health/Qatar | Government | Asia | N | N/A | N/A |
| National Comprehensive Cancer Network | Other | US | Y | Not dated | 2 |
| National Institute for Health and Care Excellence | Government | Europe | Y | 2/1/2020 | 3 |
| Royal College of Obstetricians and Gynaecologists | Professional | Europe | Y | 3/1/2020 | 2 |
| Scottish Intercollegiate Guidelines Network | Government | Europe | Y | 11/1/2019 | 1 |
| U.S. Preventive Services Task Force | Government | US | Y | 12/1/2015 | 3 |
| United States Department of Veterans Affairs and Department of Defense | Government | US | Y | 1/29/2019 | 3 |
| Wilderness Medical Society | Professional | US | Y | 10/28/2019 | 0 |
| World Health Organization | Government | Europe | Y | 1/1/2014 | 5 |
| World Society for Emergency Surgery | Europe | N | N/A | N/A | N/A |

**Data Collection Form**

The data collection form is shown below. This 25-question form was filled out by both reviewers (JHB, AED) for each potential policy document gathered in the search phase. Judgement of the policy meeting IOM standards was based on questions 12 and 13 for standard 2.1, 18 for 2.2a, 19 for 2.2b, 20 for 2.3, 23 for 2.4a, 24 for 2.4c, 25 for 2.4d

1.) Author initials:

2.) What is the full name of the organization?

3.) What type of organization is this?

a.) Professional Society

b.) Government

c.) NGO

d.) Medical Journal

e.) Healthcare Provider

f.) Other:

4.) What country/region is the organization based in?

a.) USA

b.) Canada

c.) Europe

d.) Other:

5.) If this is a US professional society, are they signed on to CMSS? (see: <https://cmss.org/membership/societies/>)

a.) Yes

b.) No

6.) Do they have a policy that addresses COI related to the production of CPGs?

a.) Yes

b.) No

7.) If there is a policy, what is the TITLE of the policy?

8.) If there is a policy, what is the DATE of the policy? (leave blank if not dated)

9.) Is COI defined in the policy?

a.) Yes

b.) No

10.) If COI is defined, what is the definition?

11.) What types of COI are considered by the policy?

a.) Financial

b.) Intellectual

c.) Professional

d.) “non-financial”

e.) Other:

12.) Are authors/members of the guideline committee required to disclose COI?

a.) Yes

b.) No

13.) If disclosure is required, WHEN are the authors required to disclose?

a.) prior to starting work OR when applying

b.) throughout time working on guideline

c.) Other:

14.) If disclosure is required, WHAT types of COI are they required to disclose?

a.) Financial

b.) Intellectual

c.) Professional

d.) Other

15.) What is the threshold (i.e. monetary value) for which financial COI must be disclosed?

16.) What is the threshold (i.e. monetary value) for which financial COI is considered disqualifying?

17) What types of financial support are explicitly required to be disclosed?

a.) Consultancy Fees

b.) Salary

c.) Research funding/grant

d.) Patent or Royalties

e.) Equity

f.) Gifts

g.) Other:

18.) Is the COI of each member required to be discussed prior to the onset of work on the guideline?

a.) Yes

b.) No

19.) Is the member required to explain how COI may affect their recommendations?

a.) Yes

b.) No

20.) Is a member with financial COI required to divest themselves of the conflicting interest prior to starting work?

a.) Yes

b.) No

21.) Who is listed as in charge of managing the COI?

22.) How is COI for a given member managed?

a.) no COI is allowed

b.) Recused from working on only relevant section

c.) Recused from voting on any section

d.) Recused from voting on only relevant section

e.) No strategy is listed

23.) Is there a statement that members should not have COI whenever possible?

a.) Yes

b.) No

24.) Is there a statement committee should not have more than 50% of members with a COI?

a.) Yes

b.) No

25.) Is there a statement requiring chairs/co-chairs to be COI free?

a.) Yes, for all chairs or co-chairs

b.) No

c.) One chair may have COI only if the other co-chair is COI-free
