# Supplementary material for: Analysis of conflict of interest policies among organizations producing clinical practice guidelines
Source: PLoS One. 2021 Apr 30;16(4):e0249267. doi: 10.1371/journal.pone.0249267 (PMC8087455; doi:10.1371/journal.pone.0249267)
Supplement: Attachment — Submitted filename: CPG COI policies Response letter_PLOS One.docx [file pone.0249267.s002.docx]

February 27, 2021

Quinn Grundy, PhD, RN

Academic Editor

*PLOS ONE*

Dr Dr. Grundy,

Thank you for the opportunity to revise our manuscript “Analysis of conflict of interest policies among organizations producing clinical practice guidelines.” We appreciate the reviewers’ insightful comments and have responded to each point in detail below. Line numbers referenced in our responses below correspond to the manuscript with tracked changes.

Sincerely,

J. Henry Brems, MD, MBE

Department of Medicine

Vanderbilt University Medical Center

**Reviewer 1**

| Reviewer Comment | Response |
| --- | --- |
| **Why were the authors expecting that the European and Canadian organizations should have policies that met the criteria of the IOM, an American body?** | We thank the reviewer for bringing up this point. Although the former IOM (now National Academy of Medicine) is an American body, its standards do not apply to only US guidelines and can be used in an assessment of any CPG. Clinicians should be wary of CPGs, regardless of where they were developed, that do not meet the IOM standards because those CPGs may be prone to inappropriate influence from COIs.  The standards have also been used in prior studies of international organizations (see citations in the revised manuscript).  Additionally, European organizations have made reference to these standards explicitly in their own documents regarding COI.  Overall, we agree this is very important context to include, so we have added to the methods on lines 146-151 with relevant supporting citations. |
| **Previous research has shown that CPGs developed by not-for-profit bodies and government agencies have fewer committee members with COI than CPGs developed by medical specialty societies and professional organizations (BMJ 2011;343:d5621). Was there a difference between these different types of organizations in this study?** | This is an important point, and one we sought to address in Tables 3 and 4. In both tables, results are stratified by professional vs governmental organizations. Table 4 demonstrates that a higher percentage of government organizations met standards 2.4c and 2.4d regarding COI among panel members and chairs/co-chairs.  We did not perform a statistical analysis to assess for statistical significance of these differences as this was beyond the scope of our study and likely would have been severely limited in the setting of a small sample size. |
| **In the Discussion, the authors should note the limitations in the IOM standards, specifically that the IOM standards apply to individual guideline committee members and not to the organizations themselves.** | We appreciate the reviewer highlighting this distinction, but we believe the IOM standards apply to the broader, organization-level development process. Individuals are not responsible for ensuring that they do not have COI or that their COI is managed. The organization (or its respective guideline development group) is responsible for ensuring these standards are met. Thus, the standards apply to the organization. |
| **In the Introduction the authors should specify how they are defining COI.** | We agree that this is conceptually important. We feel the line that the following sentence in lines 69-72 defines the specific COI we are concerned with in this manuscript:  “Conflicts of interest (COI) between the primary goal of creating optimal guidance and a committee member’s other interests, such as promoting their own research or obtaining future funding (3), however, may influence CPG development at any stage including interpretation of the evidence, drafting of recommendations, or voting on recommendations” |
| **In the Methods the authors should state whether there were language restrictions in their search.** | We thank the reviewer for noting this. We have added a phrase to the Methods on line 121 to demonstrate that we only considered CPGs published in English |
| **Table 2 lists 3 Canadian organizations and 2 "other" organizations. How are these categorized in Table 3 in terms of "region"?** | All three organizations listed as “Other” in Table 3 are Canadian. We have updated Table 3 to reflect this. |
| **Where are the Canadian organizations in Table 4?** | Although we had previously excluded this due to the small numbers, we appreciate the reviewer noting their absence. Given that the only organizations with policies are from US, Europe, or Canada, we have added Canadian organizations to Table 4. |

**Reviewer 2**

| Reviewer Comment | Response |
| --- | --- |
| **Sampling: They identified the cpgs from several sources because the clearing house has been dissolved. These organizations might not be entirely representative, however, there is a very strong argument that the sampling is adequate: The organizations were highly selected in doing at least 5 cpgs; the organization had to have explicit policies, and many did not; they had to have systematic reviews etc. Therefore, the sampling favors the hypothesis because these are among the best organizations, trying the hardest, and therefore other organizations would only be worse. The authors could emphasize this in the discussion.** | We thank the reviewer for highlighting this aspect of our study. We have expanded on this further by rewriting part of the discussion in lines 352-361 |
| **The iom cpgs standards are not “validated”; however, there have been few if any challenges, and there is redundancy so that if several standards are not met, the ones that are met would be adequate to assure at least a reasonable level of firewalls around coi. The authors do mention this in the limitations section, and they could expand on this.** | We agree and have added to the discussion on lines 367-69 |
| **In terms of remedies the authors might discuss why the clearinghouse or equivalent should be reestablished, because the pressure to not address coi is so great that only a centralized government or quasi government body can exert pressure on the organizations. I realize that this my perspective, but the clearinghouse during its existence seemed to be widely respected and many organizations attempted to conform to clearinghouse criteria.** | We agree with the reviewer that this such an independent database could exert pressure on organizations to improve management of COI. We have added to the Discussion about this on lines 323-326 |

**Reviewer 3**

| Reviewer Comment | Response |
| --- | --- |
| **Abstract: Suggested using “i) the number of” instead of “the number of i)” in line 35** | We agree and have made this change |
| **Punctuation seems to be missing at the end of the last sentence in the background section.** | We have added punctuation there on line 36 |
| **Suggested to amend line 64 "on the screening, diagnosis, prevention, and treatment of a wide variety of diseases" to "on the prevention, screening, diagnosis, and treatment of a wide variety of diseases" to make the expression more logical.** | We have made this change. |
| **If IOM standards and IOM criteria have the same meaning, Suggested uniform expression across the text. Such as lines 91, 112, 134, 228.** | We thank for the reviewer for bringing this inconsistency to our attention. “Standards” is more technically correct, so we have adjusted the language to uniformly reflect this. |
| **In line 122, suggested describing web search in detail, such as adding search engine information.** | We have added the search engine used on line 133. |
| **In lines 152 to 154, only one author reviewed each CPG and its corresponding appendices for the presence of author COI disclosures. Whether it may lead to information omission?** | We appreciate the reviewer bringing attention to this. Although it is possible a single author may have missed information, we felt the process required minimal subjectivity as author disclosures were either listed or not listed. Even if data were missed, it would mean we underestimate the frequency with which organizations violate their own standards.  As a notable limitation of our study, we have added this to the discussion in lines 372-374 |
| **Suggested providing details of Cohen's kappa calculation in the statistical methods section** | We have added further information detailing this on lines 158-160. |
| **Table 1, Suggested that the standard a/b/c after the serial number correspond to the original standard to improve the legibility of the table.** | We are unclear what the reviewer is suggesting. These are the standards as noted in the original 2011 IOM report. |
| **Suggested to add corresponding attachments for the content corresponding to lines 148 to 154 and lines 180 to 184 so that readers can understand more details.** | We are not positive what the reviewer is asking for. S1 Table in the Supplement likely includes most of this information. If there is any more specific content that we should include, please let us know and we would be happy to do so. |
| **In line 234, Suggested adding the confidence interval of Cohen’s kappa** | We have added this. |
| **If "IOM standard" in line 214 and "standard" in line 270 have the same meaning, suggested to express them uniformly.** | We thank the reviewer for noting this discrepancy. We have added “IOM” in front of “standard” on lines 296 and 297. |

**Reviewer 4**

| Reviewer Comment | Response |
| --- | --- |
| **In the abstract - it is not clear if ‘previous evidence’ (line 28/29) refers to previous studies (like Norris et al) or the IOM report** | We have changed the wording to “Studies” instead of “prior evidence” to clarify. |
| **In the introduction - the definition of CPGs as ‘documents that formulate recommendations’ could be reworded to include its purpose (i.e. clinical recommendations to optimise patient care)** | We agree this is more helpful, accurate wording. The IOM report uses part of this exact phrase in its definition so we have updated this sentence accordingly. |
| **In the introduction - consider rewording the statement in line 72 ‘Moreover, CPGs often do not have access to robust high-quality evidence…’ to ‘CPG developers often do not have access to robust high-quality evidence…’** | We agree that this is the more correct way to make this statement and have made the change accordingly. |
| **The keywords listed in manuscript does not include ‘COI’** | We have inserted this into the keywords in the manuscript. |
| **Consider adding a clear aim/objective of the study in the introduction or methods section, either in addition to the statement in line 99/100 or by rewording/expanding that statement.** | We appreciate the reviewer pushing us to make the objective of our study more clear. We have reworded the closing sentence of the introduction on lines 108-109 to clarify our objective. |
| **Suggest the authors clarify why the fact that the National Guidelines Clearinghouse is no longer available is relevant to this study (e.g. it is the database that was used by Norris et al)** | It is no longer available due to a loss of government funding. We have added a clarifying statement on line 115 |
| **It is unclear how/why the guideline databases were chosen for this study and what their potential limitations are.** | The NGC was unfortunately the primary database used previously. As there is no similarly comprehensive database to our knowledge, we searched three databases to ensure as complete of a search as possible. We have amended the methods in line 115-116 to express this. |
| **Authors can include a statement on why the seven IOM standards used in the comparison were selected (e.g. COI-specific criteria used by Norris et al)** | We thank the reviewer for highlighting this. We used these both because they are the IOM standards that can be evaluated within a policy and because they allow for comparability to Norris et al. We have added a sentence on lines 145-146. |
| **The CPG developing organisations identified (presented in Table 2) are based in the United States (n=23), Canada (n=3), Europe (n=16), Asia (1) and ‘other’ (n=3). Based on the description of the CPGs included under ‘other’ it appears that no CPG developers based in Africa or South America that met the inclusion criteria were identified. The authors may want to consider providing some thoughts on this – it may be an important finding in itself or potentially indicate a limitation of the databases searched?** | This is a subtle but important finding in our study. It is possible fewer CPGs are developed by organization based in these regions, but we cannot conclusively state that based on our results. It is an important limitation, and we have noted it in the Results on lines 198-200 and commented on in the Discussion on lines 363-364 |
| **In the Disclosure section (line 191/192), the authors state that “A significant majority of these (33, 92%) policies specified that disclosures must occur prior…” It will be helpful if the authors can explain why they considered 34% a ‘significant majority’.** | We did not write this phrase clearly enough. There were 33 policies, comprising 92% of the 36 total policies, that specified the finding referenced. To ensure clarity, we changed it to “Almost all (92%) policies…” on line 213 |
| **In the discussion section, the authors state (line 308/309) they only included ‘commercial COI’ in their assessment. This is not clearly stated in the analysis presented in the results section under ‘Management of COI’. It is mentioned in the methods section that this data was extracted.** | We thank the reviewer for highlighting distinction and lack of clarity. The specification of commercial COI was only done for the assessment of COI disclosures within CPGs to evaluate for violations of an organizations policy. Thus, it is not applicable to the results presented under the ‘Management of COI’ section. We have added a clarification on line 338 in the Discussion. |
| **The strategy for managing CPG panellists’ COI described under ‘Management of COI’ (line 208-212) only describes 33 policies, while 36 organisations had COI policies. The authors may want to consider including a description of the content of the other 3 policies as well for the sake of completeness.**  **Composition of COI panels - Clarification required on two points: - Lines 214 to 217: The authors state that 13 organisations required al chairs and co-chairs to be free of COI. However, Table 3 (last column - IOM standard 2.4d) reports the number of organisation to be 15.**  **The overall analysis (management of chair and co-chair COI) only describes 32 policies (13 required all chairs and co-chairs to be free of COI, 12 had no such requirement, 7 allowed one chair or co-chair to have a COI).** | We highly appreciate the reviewer catching this mistake. After review of the data, a typo in the formula in our Excel data sheet caused a miscalculation of the results in this paragraph as well as the paragraph below it. Table 3 has the correct number or organizations (15) listed. The correct numbers have been updated on lines 230-233 and lines 236-237. In light of this error, the rest of our calculations in Excel were reviewed and confirmed to be correct. |
| **Figure 1 – The authors could consider adding ‘IOM standards’ in the figure title or x-axis label** | We agree this would help with interpretation of Figure 1 and have adjusted the x-axis to state “No. IOM Standards Met” |
| **The numbers of CPG developing organisations with COI policies reported in Table 2 and Table 3 don’t align. For example, Table 3 reports 14 European CPG developing organisations with COI policies, while only 13 is reported in Table 2. In addition, Table 3 does not include Canada or Asia (as in Table 2). The first column of Table 3 suggests that all 36 CPGs with COI policies should be included in those numbers. Explanation for these discrepancies is required, e.g. if some of the fields were merged (e.g. US and Canada CPGs reported in Table 2 were combined and presented as US in Table 3) or the type of organisations included under the different fields changed (e.g. CPG organisations included under ‘other’).** | We thank the reviewer for pushing us to clarify this. The reason 14 European organizations are shown in Table 3 are because one of the ‘Other’ from Table 2 is a European medical journal. We have edited the subtext of Table 2 to help clarify this.  In Table 3, we have adjusted the “Other” category to specify that these were all Canadian organizations. As noted in Table 2, no Asian organizations had a COI policy, thus none are included in Table 3. We have added subtext to Table 3 to clarify the remaining “Other” category. |
| **The meaning of the second part of the following statement (line 282/283) is not clear: ‘Further improvement could be achieved through increased transparency and identification of trustworthy guidelines.’ Unclear what aspect ‘…identification of trustworthy guidelines…’ will improve. It is also unclear if the author is referring to clinical practice guidelines or COI guidelines (like the IOM standards).** | We agree with the reviewer that this statement is not clear, and we have re-written that sentence entirely on lines 307-309. |
| **Authors may want to clarify statement in line 315-318: ‘Although our study is focused on the extent to which published COI policies adhere to IOM guidelines and so does not provide evidence of how bias may affect CPG recommendations, limited data suggest that COI can undermine their objectivity. Thus, recommendation of a drug may be more likely when authors have a COI with the pharmaceutical company producing that drug.’ It is not clear if ‘limited data’ refers to data collected as part of this study, or by other studies (as mentioned in the next sentence of that paragraph – line 317/318).** | We agree this statement should be clarified. We are indeed referring to other studies as mentioned in the next sentence. We have changed the wording to specify that we mean “limited prior data.” on line 347. |
