# Supplementary material for: Analysis of conflict of interest policies among organizations producing clinical practice guidelines
Source: PLoS One. 2021 Apr 30;16(4):e0249267. doi: 10.1371/journal.pone.0249267 (PMC8087455; doi:10.1371/journal.pone.0249267)
Supplement: Attachment — Submitted filename: CPG COI policies Response letter 2_PLOS One 3.13.docx [file pone.0249267.s003.docx]

March 13, 2021

Quinn Grundy, PhD, RN

Academic Editor

*PLOS ONE*

Dear Dr. Grundy,

Thank you for the opportunity to further revise our manuscript “Analysis of conflict of interest policies among organizations producing clinical practice guidelines.” We appreciate the additional comments and have responded to each point in more detail below.

Sincerely,

J. Henry Brems, MD, MBE

Department of Medicine

Vanderbilt University Medical Center

**Comment 1 [C1]: the abstract conclusion is rather abrupt; would you consider adding a sentence or two on the implications of these findings for policy or practice?**

Response 1 [R1]: We appreciate this point and agree the conclusion could be improved. We have added two sentences to the end to further delineate the implications of our findings.

**C2:** **you did not address Reviewer 1's comments regarding clearly defining conflict of interest in the Introduction. The definition you provide on lines 67-71 cites the IOM, but only refers to non-financial interests, which is not the main focus of the IOM report. You then refer to "financial COI" (which reflects standard 2.3, which you assess) and then "commercial COI", but do not define these terms. In the discussion, you re-introduce these and "intellectual" COI, but state that you only assessed "financial" COI. Please address the use of multiple terminologies and clearly define these concepts up front. I would suggest using the IOM definition and then defining the concept in terms of what you will actually go on to assess. Introducing the "intellectual" COI prior to the discussion (and without defining it) is confusing.**

R2: We thank the reviewer for pushing us to clarify our definitions. We agree this is critical, and it is an often misunderstood aspect of COI. We have made changes to the introduction, and particularly added a paragraph to define COI, financial COI, commercial COI, noncommercial COI, and intellectual COI.

**C3: Reviewer 1 also referred to conflicts of interest that may arise at the organisational level. You may want to comment in the Discussion about whether/how these standards could be adapted to address situations where a guideline developer accepts funding from an industry with an interest in the guideline topic, for example**

R3: We appreciate the reviewer highlighting this point, since organizational level COI is also extremely relevant. Organizational COI, perhaps resulting from the acceptance of industry funding for CPG development, may significantly affect CPGs. The IOM standards do partially address this, albeit not the standards included in this study. From the 2011 IOM report, standard 1.1 states that the “processes by which a CPG is developed and funded should be detailed explicitly and publicly accessible.” Although organizational funding was beyond the scope of our study, we absolutely agree it is worth discussing the implications. As such, we have added to the discussion about this on lines 322-328 in the marked copy of the manuscript.

**C4: You comment on the generalisability of the sample and also on the geographic**

**representation, but attribute this to exclusions based on language (which applies to South America but Africa less so). You do not include any guidelines produced by or with the WHO - might this affect your sample? You also do not include any from Australia/New Zealand (e.g. National Health and Medical Research Council). Please reflect on the strengths/limitations of your sample in terms of generalisability of findings beyond language restrictions alone.**

R4: We again thank the reviewer for pushing us to clarify and reflect on this point. It certainly represents an important limitation. We suspect multiple factors may affect this. Without the NGC, we had to devise a new method to find CPGs, but as this hasn’t been used previously, we certainly may have missed some. Language may have been an impact as already noted for some, as this made it difficult, for example, to search Japanese websites for additional CPGs produced by organizations identified in our database search. Additionally, it is possible that organizations from those regions do not produce as many CPGs. For example, among the 907 CPGs reported, we identified 2 from the Australian NHMRC in the eligible time frame. We also identified one CPG each from five different Japanese CPGs. However, none of these organizations met inclusion criteria due to publishing fewer than 5 CPGs in the eligible time frame. As noted in the S1 Table in Appendix, we did include the WHO. As its headquarters are in Switzerland, we considered it a European organization.

Overall, we agree this limits the generalizability of our study, and it deserves additional attention in the discussion. We have added to the discussion on lines 373-378 in the marked copy of the manuscript.
